# Supplementary material for: The HEADS: UP Development Study: Working with Key Stakeholders to Adapt a Mindfulness-Based Stress Reduction Course for People with Anxiety and Depression after Stroke
Source: Healthcare (Basel). 2023 Jan 26;11(3):355. doi: 10.3390/healthcare11030355 (PMC9914141; doi:10.3390/healthcare11030355)
Supplement: Supplementary file 1 [file healthcare-11-00355-s001.zip › healthcare-2082666-supplementary/Supplementary file S1. Mindfulness information for participants_Oct.2017.pdf]

HEADS: UP

Helping Ease Anxiety and Depression after Stroke

You will be participating in a **HEADS: UP taster session and focus group** that is based on the eight week **Mindfulness** Based Stress Reduction course. We would like you to immerse yourself as best as you can into this process. Be curious to your experience in the moment, let go of opinions and ideas and put aside for a while any plans you may hold about applying these skills in your personal and professional life.

What is Mindfulness?

Mindfulness is a life skill that can deepen our sense of well-being and fulfilment. When we practise Mindfulness, we are in touch with ourselves, paying attention to our present experience in the moment, with an open and non-judgemental attitude. We engage all of our senses and we become aware of our body, emotions, thoughts and environment.

Mindfulness can be experienced by everyone!

You may have experienced mindfulness and not realised it. You might have felt this when you were present in places of natural beauty, simply “breathing it in,” like before a sunset or beside the sea. More often, though, our mind wanders from one thing to another. We are constantly distracted and caught in our thoughts and worries about the past and the future. This may cause stress and stress-related health problems.

Mindfulness can help us achieve a sense of brightness, clarity of purpose and inner peace.

Mindfulness helps us to change the relationship we have with our everyday problems and enables us to work with our life’s challenges.

Experiencing Mindfulness

You will experience some techniques used in Mindfulness during the taster session on [insert date].

The two Mindfulness trainers will guide you through these experiences.

You are invited to wear loose and comfortable clothing, appropriate for some gentle body movement and stretching.

We hope you will enjoy your experience of Mindfulness. At the end of the session there will be a break for refreshments. After this we will invite you to take part in a focus group and share your thoughts on the Mindfulness session.

We look forward to meeting you!
