# Supplementary material for: The HEADS: UP Development Study: Working with Key Stakeholders to Adapt a Mindfulness-Based Stress Reduction Course for People with Anxiety and Depression after Stroke
Source: Healthcare (Basel). 2023 Jan 26;11(3):355. doi: 10.3390/healthcare11030355 (PMC9914141; doi:10.3390/healthcare11030355)

HEADS: UP study- Helping Ease Anxiety and Depression after Stroke

Mindfulness Taster Session and Focus Group Information

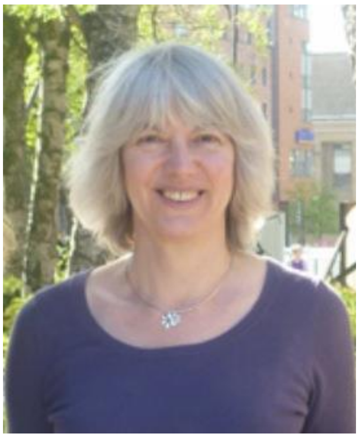

Maggie Lawrence is the Lead Researcher of this study.

Bridget Davis and Leyla De Amicis are the two researchers working on this study.

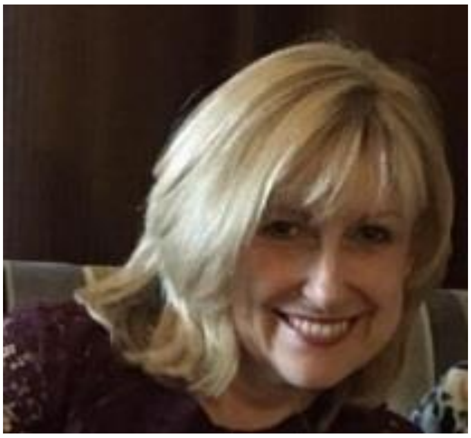

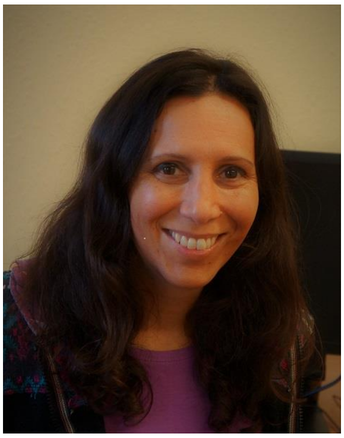

Mindfulness Taster Session and Focus Group Information

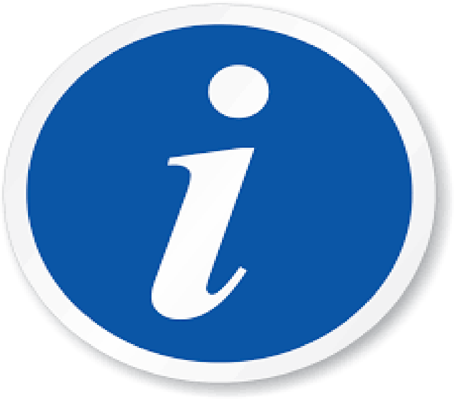

This pack will give you **information** on:

- How to walk to the CEE building at Glasgow Caledonian University
- Parking facilities
- How to get to the CEE 3 room
- Where to find the toilets
- Mindfulness brief information

Your Session

You agreed to attend the **HEADS UP** session on:

Date: **Tuesday 17th October 2017**

Start Time: **10.15** am

End time: **1** pm

Glasgow Caledonian University (GCU)

The **address** for the GCU is:

Cawcaddens Road

Glasgow

G4 0BA

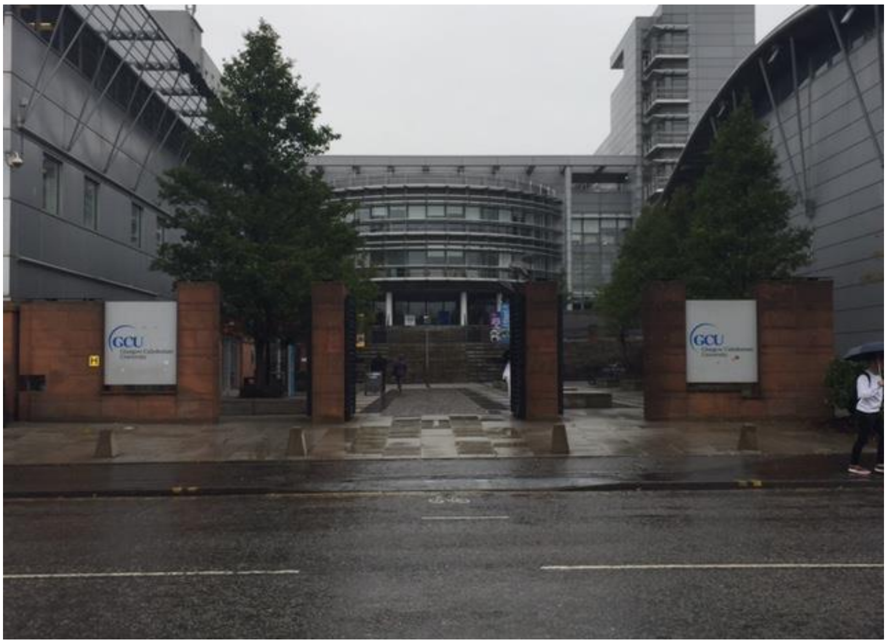

If you are walking from **Buchanan Bus Station**, the closest GCU entrance for the **CEE** building is on **Cowcaddens Road.** The picture above shows you the entrance.

There will be **signs** at the University entrance to guide you to the **CEE building.** If you need to contact us on the day, please call **07722806719** (Leyla) or **07411115765** (Bridget).

How to get to the CEE building

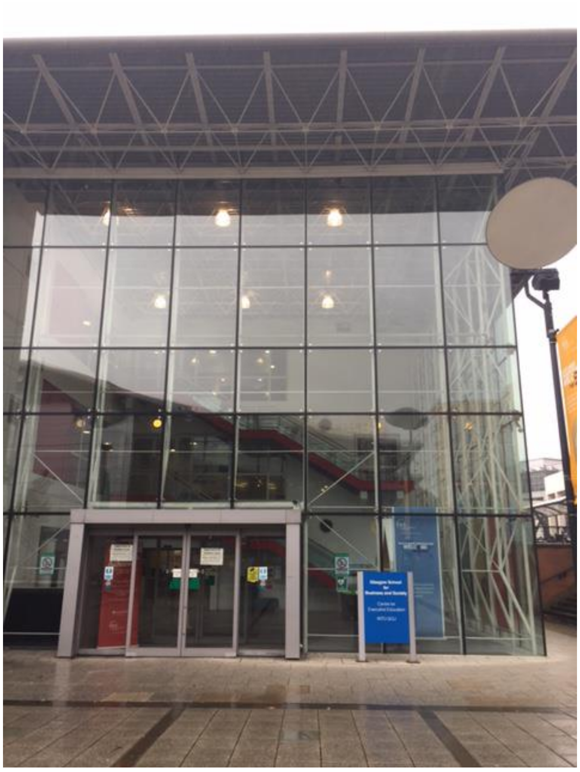

When you enter the GCU entrance close to the bookstore, you will see on your left side a Santander bank branch. The CCE building is next to it.

Opposite the **CEE building** there is the **ARC** centre.

Parking facilities

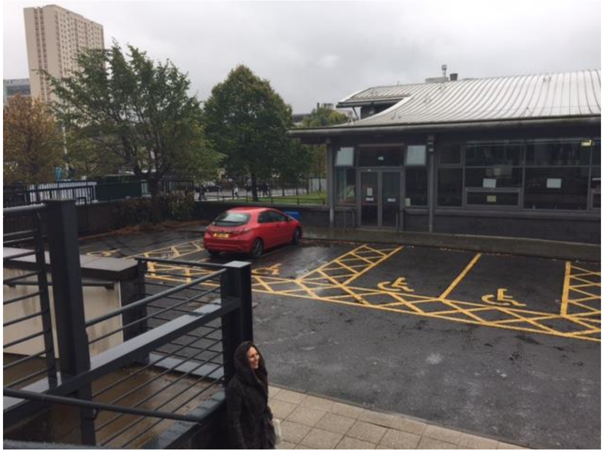

If you have a blue badge you can park in the **disabled car park**, entering from **North Hanover Street**.

This car park is through the barrier, on your left.

If you do not have a blue badge you can park in the **car park** through the barriers from **Cowcaddens Road**. You will need to say to the Security guard your **name, surname** and **"HEADS UP study"**.

How to Get to the Floor Level 1

The CEE 3 room is on **Floor Level 1**. You can take the **lift** to go to Level 1. Everything you need will be here on this level.

- When you enter the CEE main entrance, you will see on your **left** side a lift to access Level 1.
- The lift is at the end of the entrance room.
- In the picture a gentleman is waiting for the lift.
- If you prefer, you can take the **stairs**. They are on the right of the CEE main entrance.

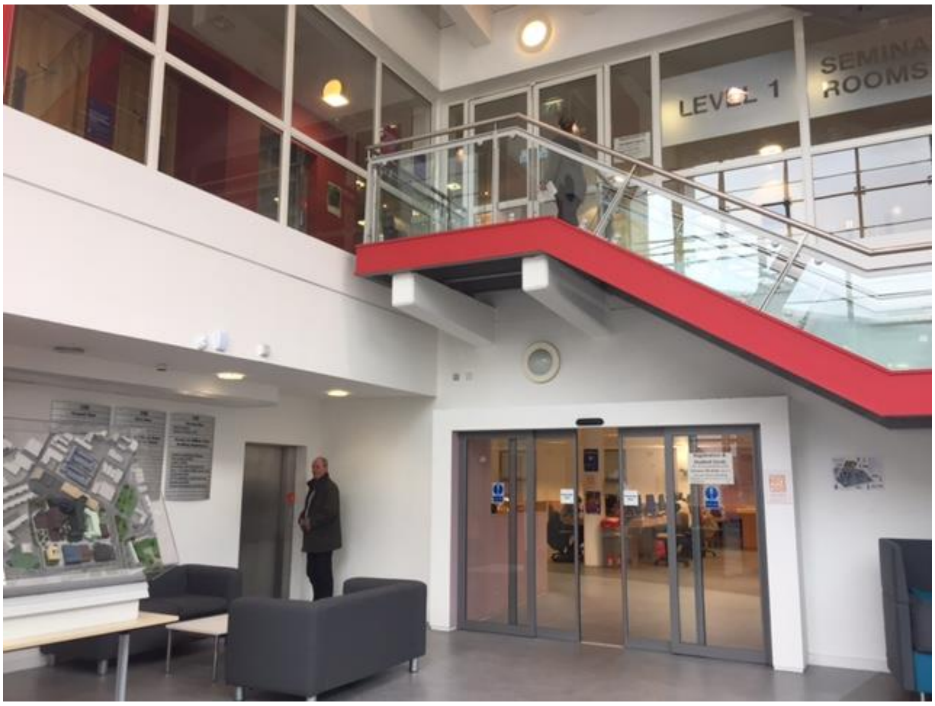

How to Get to the CEE 3 room

- When you walk out of the lift, the CEE 3 room is on your right.
- The CCE 3 room will be in front of you. In front of the CEE3 room there are some seats. This is where we will have refreshments, during the break.
- If you prefer, you can stay in the room during the break.

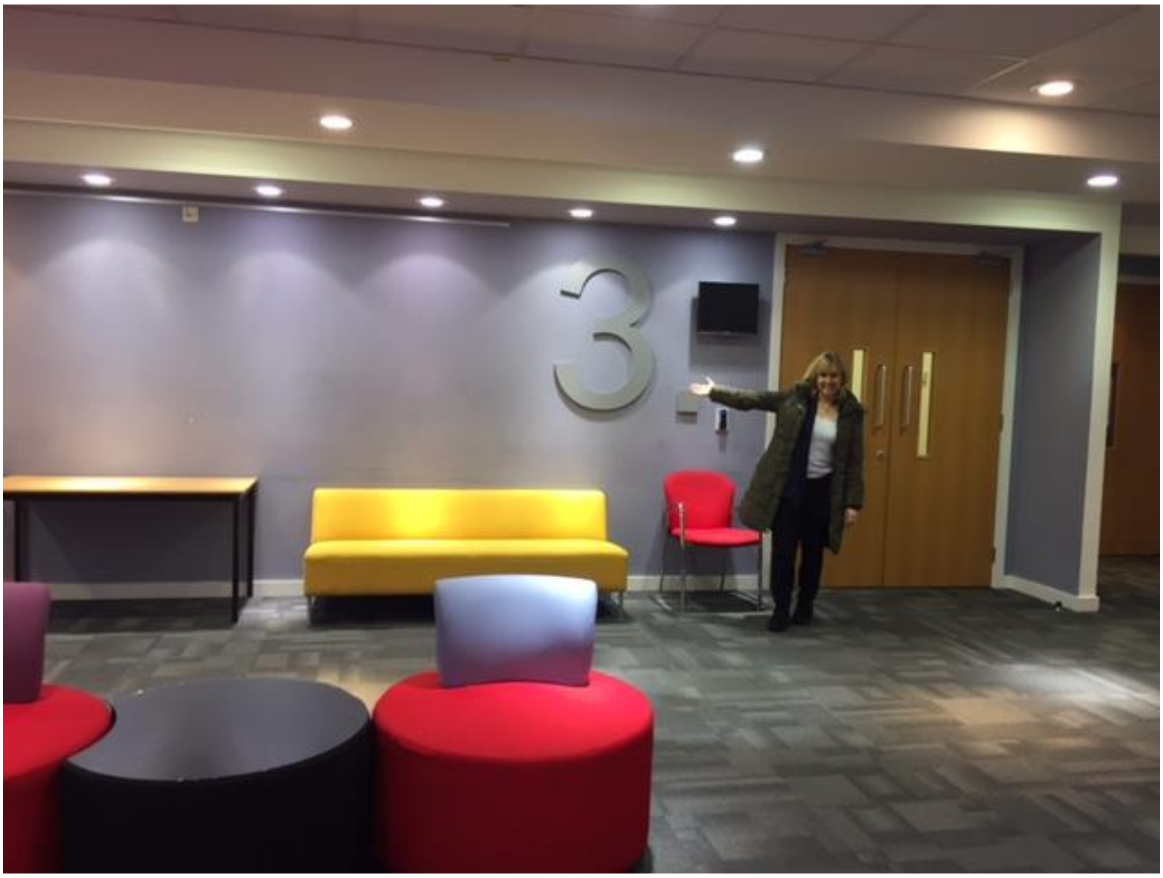

Where are the toilets?

- The toilets are close to the lift.
- When you walk out of the lift, they are on your right side.

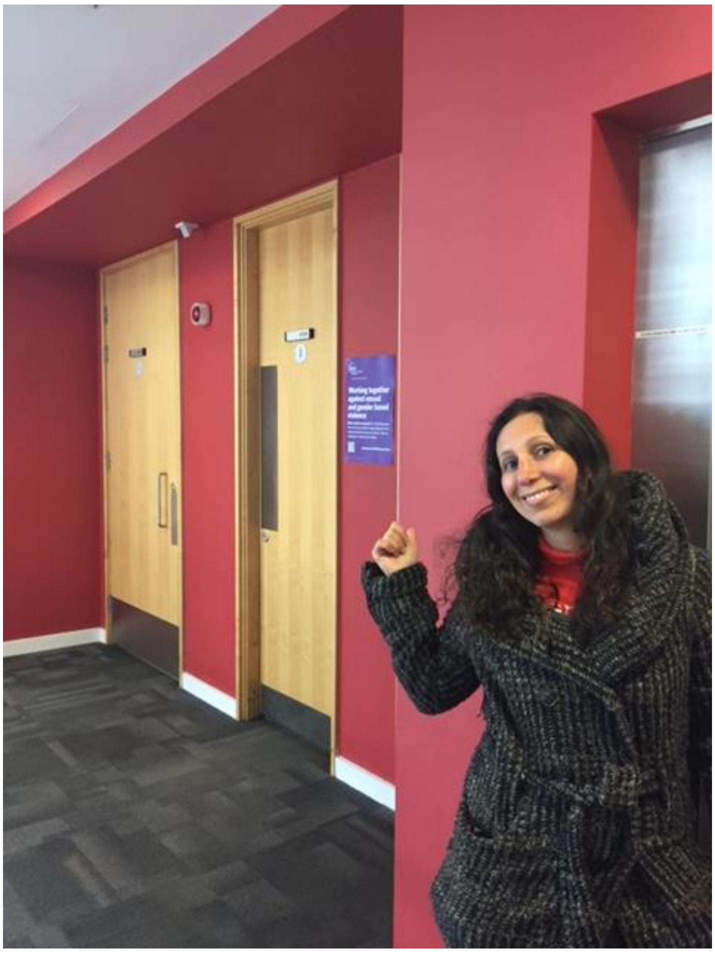

Supplement: Supplementary file 1 [file healthcare-11-00355-s001.zip › healthcare-2082666-supplementary/Supplementary file S2. Accessible Information Pack__Oct.2017.pdf]
