# Supplementary material for: The HEADS: UP Development Study: Working with Key Stakeholders to Adapt a Mindfulness-Based Stress Reduction Course for People with Anxiety and Depression after Stroke
Source: Healthcare (Basel). 2023 Jan 26;11(3):355. doi: 10.3390/healthcare11030355 (PMC9914141; doi:10.3390/healthcare11030355)
Supplement: Supplementary file 1 [file healthcare-11-00355-s001.zip › healthcare-2082666-supplementary/Supplementary file S3. Focus group schedule_April 2018.pdf]

Second focus group (April 2018)

Welcome and introductions

Focus group ground rules

Questions

What did you think about the mindfulness class this morning?

What did you think of the Information we sent out:

Did it help you to feel prepared for today?

Was it easy to understand?

What did you think of the format of the taster session

Was the group a good size/too many people/not enough people

What did you think of the trainer today? Could you understand the instructions/could you hear?

If you attended an 8 week session would prompts from the researchers (to keep in touch) be of help to you?

Would you attend with a family member/friend? How would that help you with your mindfulness experience? Would you be able to help each other with prompts?

Did anyone follow-up any of the information after the October taster session – what did you do?

new participant's thoughts (Irvine only)

What did you think about any changes you noticed?

What did you think was hard?

What did you think was easy?

Will you practice anything you learned today? Will you tell anyone else about it/encourage them to try it? If so, why?

What did you practice, if anything, after the last class/focus group?

Are there any other changes you would suggest that would make Mindfulness courses easier for stroke survivors to follow?

Any other thoughts …?

Close of focus group; reminder that research summaries will be sent to participants, if requested

Reminder about expenses claim forms/taxis

Thanks and goodbyes
